# The availability of global guidance for the promotion of women’s, newborns’, children’s and adolescents’ health and nutrition in conflicts

**DOI:** 10.1136/bmjgh-2019-002060

**Published:** 2020-11-22

**Authors:** Samira Aboubaker, Egmond Samir Evers, Loulou Kobeissi, Lauren Francis, Robinah Najjemba, Nathan P Miller, Steve Wall, Daniel Martinez, Joseph Vargas, Per Ashorn, BRANCH steering committee, Zulfiqar A Bhutta

**Affiliations:** 1 Independent Consultant, Geneva, Switzerland; 2 Department of Maternal, Newborn, Child and Adolescent Health, WHO, Geneva, Switzerland; 3 Department of Reproductive Health and Research, WHO, Geneva, Switzerland; 4 Health Section, UNICEF, New York, New York, USA; 5 Saving Newborn Lives, Save the Children, Washington, DC, USA; 6 Medical Department, MSF, Geneva, Switzerland; 7 UNHCR, Geneva, Switzerland; 8 Faculty of Medicine and Health Technology, Tampere University, Tampere, Finland

**Keywords:** nutrition, child health, maternal health, health policy, public health

## Abstract

**Background:**

Significant global gains in sexual, reproductive, maternal, newborn, child and adolescent health and nutrition (SRMNCAH&N) will be difficult unless conflict settings are adequately addressed. We aimed to determine the amount, scope and quality of publically available guidance documents, to characterise the process by which agencies develop their guidance and to identify gaps in guidance on SRMNCAH&N promotion in conflicts.

**Methods:**

We identified guidance documents published between 2008 and 2018 through English-language Internet sites of humanitarian response organisations, reviewed them for their scope and assessed their quality with the AGREE II (Appraisal of Guidelines for REsearch and Evaluation II) tool. Additionally, we interviewed 22 key informants on guidance development, dissemination processes, perceived guidance gaps and applicability.

**Findings:**

We identified 105 conflict-relevant guidance documents from 75 organisations. Of these, nine were specific to conflicts, others were applicable also to other humanitarian settings. Fifteen documents were technical normative guidelines, others were operational guides (67), descriptive documents (21) or advice on legal, human rights or ethics questions (2). Nutrition was the most addressed health topic, followed by communicable diseases and violence. The documents rated high quality in their ‘scope and purpose’ and ‘clarity of presentation’ and low for ‘rigour of development’ and ‘editorial independence’. Key informants reported end user need as the primary driver for guideline development and WHO technical guidelines as their main evidence base. Insufficient local contextualisation, lack of inter-agency coordination and lack of systematic implementation were considered problems in guideline development. Several guidance gaps were noted, including abortion care, newborn care, early child development, mental health, adolescent health beyond sexual and reproductive health and non-communicable diseases.

**Interpretation:**

Organisations are motivated and actively producing guidance for SRMNCAH&N promotion in humanitarian settings, but few documents address conflicts specifically and there are important guidance gaps. Improved inter-organisation collaboration for guidance on SRMNCAH&N promotion in conflicts and other humanitarian settings is needed.

**Key questions**

**What is already known?**

- Addressing the health needs of women, newborns, children and adolescents in conflicts and other humanitarian emergencies is recognised as an important global health priority.
- Significant global gains in sexual, reproductive, maternal, newborn, child and adolescent health and nutrition (SRMNCAH&N) require interventions that specifically address affected populations in conflict settings.

**What are the new findings?**

- SRMNCAH&N guidance for conflict settings exists from different organisations. However, there are important gaps in areas such as emergency contraception, newborn health, child development and adolescent health beyond sexual and reproductive health and non-communicable disease.
- There are weaknesses in the documented rigour of development and the adaptability of guidance to local contexts.

**What do the new findings imply?**

- There is need for adaptable, rigorously developed guidance that is coordinated from the development phase onwards for SRMNCAH&N in conflict settings.

## Introduction

Women and children represent the majority of populations affected by conflicts worldwide. According to an international estimate, 368 million children aged under 18 years (16% of all children in the world) and 265 million adult women (7%) were living in the proximity of armed conflict at the end of 2017.^1^ Additionally, there were approximately 36 million children and 16 million women who had been displaced from their homes due to armed conflict. Although the exact contribution of conflict to women’s and children’s health is difficult to estimate, it is evident that these vulnerable groups are disproportionally affected and that conflicts account for a large share of sexual, reproductive, maternal, child and adolescent ill health worldwide.^2–4^ In addition, the negative impacts of conflicts extend far beyond the acute phase and epicentre of the problem.^5^ In an analysis covering African conflicts between 1995 and 2015, increased child mortality was documented for 8 years after and 50 km away from the actual aggression, and most of the additional deaths were not combat related.^6^

Given the burden of conflicts, achieving significant global gains in sexual, reproductive, maternal, newborn, child and adolescent health and nutrition (SRMNCAH&N) requires interventions that specifically address affected populations in such settings. To design and deliver appropriate interventions decision makers need guidance on health promotion and service delivery in conditions where populations are displaced, where people live in temporary shelters in adverse environments, their security and access to healthcare is reduced and health system may be grossly disrupted. The World Health Organization (WHO) has the mandate of producing technical guidance for health for its member states, but thus far most of such guidance has not addressed conflicts or other emergency contexts. In recent years, WHO and other actors have developed or adapted guidance documents for humanitarian emergencies, as illustrated in the Sphere handbook.^7^ It remains unclear, however, how well existing guidance meets the needs of implementing agencies and individuals operating in conflict areas or other humanitarian emergencies.

In this paper, we describe results of a review of public guidance documents for promoting SRMNCAH&N in conflicts. Our main objective was to determine the scope and quality of currently available global guidance, the lead development agency, document type, intended beneficiary and the addressed health topic. We supplemented this review with key informant interviews to characterise the process by which agencies typically develop or update their guidance documents and ensure their dissemination and implementation.

## Methods

### General approach

The study was conducted in three phases. In the first phase, we identified guidance documents for inclusion in our review. In the second phase, we reviewed the identified documents for their scope and assessed them for their content and quality. In the third phase, we conducted key informant interviews with representatives from implementation agencies working in conflicts and other humanitarian settings. The key informant interviews aimed to document guidance development processes and shed light onto the decision-making process, development, dissemination and uptake.

### Availability of guidance for promoting SRMNCAH&H in conflict situations

For the purpose of this article, we used the WHO definition of a ‘guideline’: a document containing recommendations for clinical practice or public health policy that informs the intended end-user on what to do in specific situations to achieve the best health outcomes possible, individually or collectively. According to this definition, a guideline offers choice among different interventions or measures that have an anticipated positive impact on health and implications for the use of resources.^8^ The WHO guideline development process is described in detail in the WHO Handbook for Guideline Development.^9^ By ‘guidance’ we refer to all advice that does not necessarily take a form of a document, nor describe alternative interventions or their comparison.

We searched for all publicly available guidance documents. To do this, we did a manual review of English language websites of all organisations that are members of the Global Health Cluster (GHC), the Global Nutrition Cluster (GNC) or the Inter-Agency Working Group on Reproductive Health in Crises (IAWG). In this screening search, we looked for all documents that applied to humanitarian emergencies, not limited to those that were labelled as conflict-related. We did this because documents that apply to conflicts are often intended to apply also to other types of humanitarian contexts. For websites with extensive content (such as those for United Nations (UN) organisations), we used their own search functions and the key words ‘emergency’, ‘humanitarian’, ‘conflict’, ‘disaster’ and ‘outbreak’.

We then filtered for documents that were published before October 2018 (the time of our review), that addressed health promotion and that mentioned at least one SRMNCAH or nutrition-related term in the title, table of content, executive summary or introduction. (online supplemental table 1). We excluded documents that addressed only natural disasters and were not applicable for conflict settings (judged subjectively through document review), that offered no substantive recommendations, or were published only as scientific reports or as training modules. Last, we excluded documents published before 2008. This time cut-off was chosen primarily because it marked the period when WHO standardised its guidance development process. Limiting the review period to the latest decade also increased the topicality of the findings and increased the possibility of interviewing individuals who had been involved in developing the document. When multiple versions of a document existed, only the most recent version was considered.

10.1136/bmjgh-2019-002060.supp1 Supplementary data

Two reviewers identified the documents, conducted content analysis using jointly agreed criteria (online supplemental table 2) and reconciled differences in categorisation. They classified documents as ‘technical normative’, ‘operational’, ‘descriptive’, ‘legal, human rights or ethics’ or ‘other’. We considered the organisation or network that was indicated on the first page or acknowledgement of a document as the lead agency for document development. Our primary approach was for guidance documents to self-identify the intended beneficiary group, but if this was not provided, the two document reviewers classified the intended beneficiary group into the following categories: women, newborns, children or adolescents. Similarly, we classified the addressed health topic or technology into the categories of sexual and reproductive health, pregnancy and perinatal care, immunisation, communicable diseases and infections, non-communicable diseases (NCDs), mental health and child development, injury and trauma, violence, nutrition or other. Finally, we categorised the target audience as individual, family, first level health worker, hospital professional, programme manager or not applicable/other, and checked whether the document made special reference to reduced resources, reduced access to care or other disruptions in health system during conflicts. For the intended beneficiary group, addressed health topic or technology, and target audience, multiple options were possible.

10.1136/bmjgh-2019-002060.supp2 Supplementary data

### Assessment of the quality of the available guidance documents

To assess the quality of available guidance, we used the ‘Appraisal of Guidelines for REsearch and Evaluation II (AGREE II)’ tool.^10^ This is a widely used assessment instrument for guidelines, with a total of 23 questions addressing six quality domains: ‘Scope and purpose’, ‘Stakeholder involvement’, ‘Rigour of development’, ‘Clarity of presentation’, ‘Applicability’ and ‘Editorial independence’. It uses a 7-point Likert Scale, ranging from 1 (Strongly Disagree) to 7 (Strongly Agree).

Two reviewers independently reviewed all documents, following the AGREE II tool online training instructions and Users’ Manual guidance. When an item was unclear, the reviewers sought advice from a third person with extensive experience in use of the tool. Using the values from both reviewers, we calculated six domain-specific scores and expressed the obtained score as a percentage of the theoretical maximum for that domain. As recommended in the AGREE II instructions and in the absence of a validated mathematical formula, we did not combine domain-specific scores into any overall scores. We also did not subjectively allocate any overall quality value for individual documents.

We assessed agreement between the two reviewers by calculating Spearman’s correlation coefficients for the scores they gave, overall and separately for each of the six AGREE II domains.

### Process for guidance development, dissemination and uptake

We used key informant interviews to collect information on the process of guidance development, dissemination and uptake and to identify perceived gaps in guidance for SRMNCAH&N promotion. We interviewed experienced experts from major organisations involved in humanitarian work in conflicts, using purposive sampling for organisation selection (online supplemental table 3). During sampling, we focussed on including the main organisations that were involved in developing the reviewed guidance documents: we ensured diversity of inclusion to achieve saturation in data and response. Within each organisation, we primarily selected the respondent ourselves, based on personal knowledge of the intended respondent’s professional background and experience or a clear indication on the organisation’s website of his or her role. Where necessary, we asked for alternative respondents from the initially selected individual. Interviewees were briefed on the objective of the interview and the confidentiality of the their individual responses. Interviewee consent was sought verbally and documented in the interview notes before proceeding with the interview.

10.1136/bmjgh-2019-002060.supp3 Supplementary data

We conducted the interviews using a semi-structured interview guide (online supplemental table 4), with open-ended questions that allowed flexibility and dialogue on topics that the respondents considered important. The interview guide was developed after a preliminary analysis of available guidance and its quality. This allowed a focus on key aspects of guidance development in different agencies.

10.1136/bmjgh-2019-002060.supp4 Supplementary data

One experienced professional with qualitative research experience and knowledge of guidance development and humanitarian contexts conducted all the interviews, primarily in person, but, when this was not possible, by Skype call. The face-to-face interviews were electronically recorded with a mobile phone and transcribed word-by-word to written documents, after seeking consent for recording. The Skype discussions were recorded in written notes. The same researcher who conducted the interviews also analysed the results. This was done by marking selected themes in the transcripts and notes by hand and summarising them by topic.

### Identification of gaps in guidance

We used two approaches to identify gaps: the document review and key informant interviews. For the document review, health topics or beneficiary groups that were under-represented in document numbers were considered to be potential guidance gaps. From the key informant interviews, we considered items mentioned by the interviewees as possible gaps, without consideration of how often they were mentioned. Our own conclusion about gaps was based on the topic or beneficiary group being identified by either of these two approaches.

## Results

### Scope of guidance for SRMNCAH&N in conflict settings

We searched websites of a total of 75 organisations and networks (online supplemental table 5) and identified 194 documents that potentially provided guidance on SRMNCAH&N promotion in any humanitarian emergency. On an initial review, 89 documents were deemed non-applicable to conflicts or were excluded based on other criteria. The remaining 105 documents were included in the analysis and are listed in online supplemental table 6), with hyperlinks to the actual publications.

10.1136/bmjgh-2019-002060.supp5 Supplementary data

10.1136/bmjgh-2019-002060.supp6 Supplementary data

Of the 105 included documents, only 9 (8.6%) were specific solely to conflicts, while the others were also applicable to other emergency settings. In justifying the need or content of the offered guidance, 70 documents made special reference to reduced resources, 47 mentioned reduced access to care due to insecurity/safety and 56 referred to disruptions in health service delivery.

Sixty-three per cent of the documents (66/105) were operational guides targeting programme managers. Only 14% (15/105) of the documents were classified as technical normative guidelines targeting field level health workers (tables 1 and 2). Sixty-six per cent (69/105) of the documents were developed in the last 5 years (2013 to 2018), with the largest number (16) developed in 2014.

**Table 1:** Number of identified guidance documents, by the type of document and the lead organisation for document development

Document type*	Number of documents by the lead organisation for development				
	WHO	Other UN organisation	INGOs	Other†	All together
Normative technical guidelines	8	2	5	0	15
Operational guides	6	11	23	27	67
Advice on legal, human rights or ethics questions	0	0	2	0	2
Descriptive documents	5	4	4	8	21
Other documents	0	0	0	0	0
All documents together	19	17	34	35	105

*Document categorisation is explained in detail in online supplemental table 2.

†Includes inter-agency networks.

INGO, international non-governmental organisation; UN, United Nations.

**Table 2:** Number of identified guidance documents, by the addressed health topic or technology and intended beneficiary group

Main addressed health topic or technology	Number of documents by the intended beneficiary groups				
	Women	Newborns	Children	Adolescents	All together
Sexual and reproductive health	17*	4	3	11	18
Pregnancy and perinatal care	8	6	2	1	10
Immunisations	2	3	5	0	7
Communicable diseases and infections	17	11	25	7	36
Non-communicable diseases	1	3	1	0	3
Mental health and child development	6	5	15	6	18
Injuries and trauma	3	2	7	1	9
Violence (including sexual violence)	15	3	11	13	25
Nutrition	9	15	36	2	38
Other	2	7	7	1	10
All documents together	46	22	69	32	105

*Numbers are not mutually exclusive, that is, the same document may be considered in different rows or columns, if it addresses several topics or beneficiary groups.

The document development was most frequently led by the WHO (19 documents). For 17 documents, the development was led by other UN agencies (some in combination with the WHO), 34 by international non-governmental organisations (NGOs) and 35 by inter-agency networks, other lead agencies or combinations. A detailed analysis of the numbers of different lead organisations and types of guidance documents they developed is shown in table 1.

Forty-six of the documents (44%) contained recommendations concerning women’s health, 22 (21%) addressed newborns, 69 (66%) concerned children and 32 (30%) were for adolescent health. The most commonly addressed health topic was nutrition, followed by communicable diseases, violence (including sexual violence), mental health and child development and sexual and reproductive health. There were very few documents relating to NCDs. A more detailed analysis of the numbers of documents addressing different topics and beneficiary groups is shown in table 2 and online supplemental table 7.

10.1136/bmjgh-2019-002060.supp7 Supplementary data

Most of the documents covering women’s health addressed sexual and reproductive health, communicable disease and violence and were primarily for programme managers.

For newborns, most of the available guidance concerned communicable diseases and nutrition. The focus was strongest on promoting breast feeding and on preventing perinatal HIV transmission. There was also information on containing outbreaks. Most of the guidance was operational, targeting decision makers and implementers. Only one document addressed the general promotion of child health in conflict situations.

Guidance that targeted adolescents was scarce, and addressed almost exclusively sexual and reproductive health, or violence and injury. Only two documents referred to the special nutritional needs of adolescents, and none addressed NCDs.

### Quality of global guidance for SRMNCAH and nutrition in conflict situations

The highest aggregated AGREE II scores were in the domains of ‘scope and purpose’ (84%), and ‘clarity of presentation’ (71%). These were followed by ‘applicability’ (45%) and ‘stakeholder involvement’ (52%). The lowest scores were for ‘rigour of development’ (23%) and ‘editorial independence’ (12%).

Overall, guidance documents scored highly in description of the guidance objective(s) (mean 6.2 out of a possible 7 points), the specific health questions that were covered (6.2) and the population to which they applied (5.7). The lowest scores were for the search and evaluation of evidence (means of 1.3, 1.2 and 1.4), the description of methods for formulating recommendations (2.5), the use of external review (1.9), the provision of details about updates and funding (2.1) and the declaration of competing interests (2.1 and 1.2) (online supplemental table 8).

10.1136/bmjgh-2019-002060.supp8 Supplementary data

Normative technical guidelines received slightly higher mean AGREE II scores than the other document types, except in the domains of ‘Stakeholder involvement’ and ‘Applicability’ (table 3). In other comparisons, there were minimal differences in the mean AGREE II scores between documents developed by different organisations (table 4), those targeting different intended beneficiary groups (online supplemental table 9) or addressing different health topics or technologies (online supplemental table 10).

10.1136/bmjgh-2019-002060.supp9 Supplementary data

10.1136/bmjgh-2019-002060.supp10 Supplementary data

**Table 3:** Mean AGREE II scores for the six domains of document quality, by the type of guidance document

Document type	Number of documents	Mean AGREE II scores for the six domains of document quality					
		Scope and purpose	Stakeholder involvement	Rigour of development	Clarity of presentation	Applicability	Editorial independence
Normative technical guidelines	15	95%	52%	31%	87%	40%	19%
Operational guides	67	84%	58%	23%	70%	51%	13%
Descriptive documents	21	77%	30%	20%	63%	27%	5%
Advice on legal, human rights or ethics questions	2	69%	49%	22%	60%	36%	10%
All documents together	105	84%	52%	23%	71%	44%	12%

AGREE II, Appraisal of Guidelines for Research and Evaluation II.

**Table 4:** Mean AGREE II scores for the six domains of document quality, by the lead organisation for document development

Lead development organisation	Number of documents	Mean AGREE II scores for the six domains of document quality					
		Scope and purpose	Stakeholder involvement	Rigour of development	Clarity of presentation	Applicability	Editorial independence
WHO	19	85%	50%	28%	75%	38%	14%
Other (multiple) United Nations organisations	17	85%	50%	26%	77%	43%	15%
International NGOs*	34	84%	53%	21%	69%	46%	12%
Other†	35	81%	52%	22%	68%	47%	9%
All documents together	105	84%	52%	23%	71%	45%	12%

*Non-govenmental organisation.

†includes inter-agency networks and combinations of different organisation types.

AGREE II, Appraisal of Guidelines for Research and Evaluation II.

For all domains combined, the inter-assessor agreement of document quality was excellent, as indicated by correlation coefficient of 0.90. For each individual domain, the inter-assessor agreement was good-to-excellent as indicated by correlation coefficient of 0.71 for ‘Scope and purpose’, 0.89 for ‘Stakeholder involvement’, 0.86 for ‘Rigour of development’, 0.78 for ‘Clarity of presentation’, 0.72 for ‘Applicability’ and 0.99 for ‘Editorial independence’.

#### Process for guidance development and distribution

Out of the 14 organisations identified for key informant interviews, 13 had one or more employees who were involved in guideline development, were available and agreed to be interviewed (online supplemental table 3). The organisations included major UN agencies (WHO, United Nations Children’s Fund (UNICEF), United Nations Family and Population Association (UNFPA), United Nations High Commissioner for Refugees (UNHCR)), United Nations Office for the Coordination of Humanitarian Affairs (OCHA), Inter-Agency networks (Interagency Working Group for Sexual and Reproductive Health (IAWG) and Emergency Nutrition Network (ENN)) and NGOs, including Red Cross Red Crescent Movement, World Vision, Médecins Sans Frontières (MSF), Save the Children (SCF), International Committee for Red Cross (ICRC), International Rescue Committee (IRC) and a donor agency (European Civil Protection and Humanitarian Aid Operations (ECHO). We interviewed a total of 22 experts, all of whom were knowledgeable about the full process of guidance development and implementation. The interview sessions lasted an average of 57 min, with a range of 40 to 65 min. Half of the interviews were conducted face-to-face, half through Skype connection.

All key informants emphasised the primary use of existing national guidelines if available. When these were not available or were too context-specific, the organisations typically preferred to develop guidance documents jointly with the WHO or through inter-agency collaboration, such as the nutrition coalition, ENN or the IAWG. Only as a third option did the organisations choose to develop their own guidance. In these cases, there was rarely systematic consultation across organisations to avoid duplication and facilitate coordination. Across all agencies the decision-making on a new document was based on an emerging health priority or generation of new evidence and the expressed need for guidance from end users. As one interviewee put it, ‘Decision making is organic and based on needs’.

WHO representatives indicated that their organisation followed a systematic method for developing guidance. The method includes systematic search strategies, synthesis and quality assessment of the best available evidence to support a recommendation, and the engagement of experts including content experts, methodologists, target users and policymakers. Experts are selected to ensure gender and geographical balance, and there is a mechanism to achieve consensus among experts that includes a transparent decision-making process taking into account potential harms and benefits, end users’ values and preferences. While it is solid in its way of generating evidence-based global guidance, the WHO representatives felt that the process often takes a long time and requires an additional step of adaptation or contextualisation before application in a country context. WHO representatives further indicated that the need for a faster guidance development process for humanitarian settings had been recognised at the WHO. As a result, WHO had developed a streamlined procedure through which it could smoothly produce derivative guidance from existing technical guidelines, with an abbreviated but still rigorous process.

Other organisations indicated that they typically built on WHO guidelines in health guidance but established their own working groups of internal and external experts. Often a consultant was hired to lead the development process. While acknowledging WHO’s evidence base, some of the major NGOs found WHO recommendations often too generic or outdated, making them unsuitable for practical healthcare providers. For some NGOs, scientific evidence alone was not enough for formulating recommendations, and ‘there needed to be a balance between practical experience and academics’. Consensus and field experience were considered critical in guidance development in the humanitarian field. One key informant gave an example of this position: ‘The field cannot wait for randomised controlled trials’.

The weakest reported aspects of SRMNCAH&N guidance were document dissemination, and update and monitoring of guidance uptake. Except for MSF, no organisation reported a standard protocol for document dissemination or for monitoring adherence to its recommendations. Typically, there was a plan to update each document at 3 to 5 year intervals, but this was reported to take place rarely. To disseminate documents and get feedback on their use, organisations used a variety of means including organisational websites, mailing lists, internal training or other meetings, field visits, distribution to country offices and field coordinators, international meetings and statistics on guidance requests from ‘the field’. MSF reported following a standardised model, in which information on new guidance was passed through the organisation, with clear responsibilities for acknowledging the new information and updating field guides accordingly. The MSF representative also reported that the organisation used its telemedicine programme and regular programme reviews to assess and verify adherence to its guidance. The key informant from MSF said: “Most programmes in the field are visited at least once a year. When we send consultants to the field, we also ask that they check if our guidance is used or not”.

The main guidance gaps identified by the key informants fell into three broad categories: (1) important but unaddressed health needs, (2) insufficient contextualisation and (3) consideration of the first-level health workers. In the first category respondents listed newborn care, early child development, mental health, adolescent health beyond sexual and reproductive health, adolescent male to male violence, NCDs, migrant health and the health needs of children aged 5 to 9 years. The respondents also highlighted gaps in guidance for the provision and procurement of emergency contraception and for safe abortion care. There was also a perception that little guidance existed on the comprehensive promotion of child health in conflicts or other humanitarian settings and that the available guidance was scattered in small bits. In the second category, respondents felt there was enough general guidance targeting programme managers and decision makers, but too few documents considered local context and provided contextualised advice. In a respondent’s own words “Our guidance is borrowed. We are taking the interventions delivered in a non-humanitarian setting and we implant them in a humanitarian setting. We treat Aleppo the same way we treat rural Tanzania”.

In the third category, hands-on practical advice on actual service delivery was felt to be missing. This was well illustrated in a statement from one key informant: “We may have guidance, but what is missing is a simple standardised user-friendly algorithmic type of clinical guidance”.

Key informants also identified the lack of a widely recognised and operational common platform for developing guidance for all important beneficiaries. For more than 20 years, a network called IAWG has served as a forum for identifying guidance needs and coordinating guidance development for Sexual and reproductive health (SRH) promotion in various humanitarian settings. For the promotion of newborn, child and adolescent health, no similar broad platform exists, and several respondents identified this as a hindrance for guidance prioritisation, development and health promotion for these target groups.

## Discussion

Our objective was to determine the coverage and quality of currently available global guidance for the promotion of SRMNCAH&N in conflicts. From assessment of publicly available documents and key informant interviews, we noted that such guidance is available, but there are important gaps in it and only few documents provide normative guidance specifically for conflict settings. Where guidance exists, the documents typically explain well their scope, but their evidence-base and use of external experts appears more limited. There is also insufficient contextualisation and advice on how to translate global recommendations into practical actions and organisations do not typically follow standard procedures for guideline development, distribution, updates or uptake monitoring.

Our study has some limitations that might have biassed the results. The review of documents was limited to manual Internet search of organisations from three large networks of humanitarian actors. Interviews and guidance review were conducted in English. Stakeholders interviewed included only major international actors and only guidance produced after 2007 was reviewed. Thus, some important documents may have been missed, especially if produced by organisations that do not participate in collaborative networks, are largely local/regional or are primarily operating in other fields than humanitarian health. We also used two different ways of conducting and transcribing the expert interviews (face-to-face and virtual). Furthermore, the AGREE II tool is subjective in nature for some components. However, the triangulation of information from multiple sources, and the consistent feedback that we received through the in-depth interviews suggested that there were no major omissions in our document search and data interpretation. We therefore feel that our sample findings and conclusions reliably capture the limited availability and quality of the current guidance on SRMNCAH&N in conflict settings.

Addressing the health needs of women, mothers, newborns, children and adolescents in conflicts and other humanitarian emergencies is being recognised as an important global health priority.^11^ While rigorous research can be difficult in conflict situations and in humanitarian settings in general, and evidence is at times limited,^12^ researchers are increasingly generating methods and evidence that could be translated into effective SRMNCAH&N programmes, also in conflict contexts. We are not aware of prior reviews that have examined whether and how such scientific evidence has been converted into technical or operational guidance for health actors. Therefore, we are not in a position to compare our results to those from other studies.

Our analyses identified important perceived gaps in guidance especially in provision and procurement of emergency contraception, safe abortion care, newborn care, early child development, mental health and adolescent health beyond sexual and reproductive health and NCDs. There were also only few documents addressing health promotion for migrant people or children who were older than 5 years but not yet adolescents. There are likely various, interlinking and complex reasons for these gaps. Some areas with more evidence, such as nutrition, also appear to have more guidelines.^12^ Decision-making in guidance development is not necessarily based on a systematic assessment of needs but appears to be a result of emerging priorities or generation of new evidence or recommendations on a specific topic. Donor priorities and availability of funds also appear to be a factor. It is recognised that funding for areas such as NCD, mental health and adolescent health is scarce, even where successful interventions are available. This may in part be due to stigmatisation, lack of information about cost-effectiveness of interventions or misconceptions on the urgency to address such issues.^13^

In addition to the identified gaps in specific SRMNCAH&N topics, there seems to be a widely shared perception of insufficient contextualisation of technical guidance and a lack of a holistic and multisectoral view of health in humanitarian settings. While global guidance is by its nature often general, practical considerations should be a major component, in particular in humanitarian settings.^14^ Advocacy and translation into practice at local level are recognised as essential steps.^15^ The perceived lack of practical applicability and support for uptake are expected to limit the use of the guidance that does exist. During crises, women, adolescents and children face specific life-threatening risks, including malnutrition, separation from their families, trafficking, recruitment into armed groups and physical or sexual violence and abuse, all of which require immediate action and flexible strategies.

Collaboration across sectors outside the traditional health space is essential to achieve meaningful impact. The IAWG brings together actors working in humanitarian response relating to reproductive health, including newborn health. For child and adolescent health, however, no similar forum is active to allow relevant stakeholders to share experiences and coordinate activities, such as guideline development and implementation. This role could be taken on by an existing partnership, for example by expanding the area of work of IAWG or the Partnership for Maternal, Newborn and Child Health (PMNCH), which already is tasked with coordinating MNCH efforts across organisations. Alternatively, this role could be played by the WHO in its role as the IASC Global Cluster Lead Agency for health in emergency settings and overall as the UN agency mandated to ‘act as the directing and coordinating authority on international health work’ and ‘to promote maternal and child health and welfare and foster the ability to live harmoniously in a changing total environment’.^16^ Whichever mechanism is chosen, governments and donor agencies are key to empowering this mechanism towards coordinated, high-quality, evidence-based guidance development and to understand and promote uptake and implementation.

Taken together, our study indicates that organisations involved in humanitarian response are motivated and actively producing guidance on SRMNCAH&N for conflict settings. There are, however, important perceived gaps in terms of the topics, practicality, availability and development process of existing guidance and further work involving strong inter-agency collaboration is needed. We hope that this review, including a full listing and hyperlinks to the currently available guidance documents (online supplemental table 6), will facilitate this process.

**Recommendations**

There are gaps in guidance for the promotion of sexual, reproductive, maternal, newborn child and adolescent health and nutrition in conflict settings. The international community should establish a joint platform for coordinating inter-organisational collaboration, development of relevant and adaptable guidance and for making the available guidance easily accessible to implementing partners.
