# Supplementary material for: The availability of global guidance for the promotion of women’s, newborns’, children’s and adolescents’ health and nutrition in conflicts
Source: BMJ Glob Health. 2020 Nov 22;5(Suppl 1):e002060. doi: 10.1136/bmjgh-2019-002060 (PMC7684670; doi:10.1136/bmjgh-2019-002060)
Supplement: Supplementary data [file bmjgh-2019-002060supp001.pdf]

Supplementary Table 1. Search terms for identifying documents for further review

**Inclusion terms[1] for different beneficiary groups and topics**

| **Sexual & reproductive** | **Maternal / pregnancy** | **Newborn** | **Child** | **Adolescent** | **Nutrition** |
|---|---|---|---|---|---|
| Sexual | Maternal | Neonatal | Child | Adolescent | Nutrition[1] |
| Reproduction | Mother | Neonates | Children | Adolescents | Feeding |
| Reproducing | Mothers | Newborn | Infant | Teenage | Food |
| Reproductive | Pregnancy | Babies | Young child | Teen | Undernutrition |
| Contraception | Delivery | PNC[3] | Toddler | Youth | Malnutrition |
| Family planning | Birth | | | Young people | Malnourishment |
| Sex | ANC[2] | | | | SAM[4] |
| Sexual violence | | | | | MAM[5] |
| Rape | | | | | Breastfeeding |
| Gender-based violence | | | | | Lactation |
| Post-exposure prophylaxis | | | | | |
| Abortion | | | | | |

[1]Documents including at least one of these terms in the title, table of content, executive summary or introduction were selected for further review, [2]Antenatal care, [3]Postnatal care, [4]Severe acute malnutrition, [5]Moderate acute malnutrition
