# Supplementary material for: The availability of global guidance for the promotion of women’s, newborns’, children’s and adolescents’ health and nutrition in conflicts
Source: BMJ Glob Health. 2020 Nov 22;5(Suppl 1):e002060. doi: 10.1136/bmjgh-2019-002060 (PMC7684670; doi:10.1136/bmjgh-2019-002060)
Supplement: Supplementary data [file bmjgh-2019-002060supp002.pdf]

Supplementary Table 2 - Categorization criteria for the reviewed documents

We classified the guidance reviewed by document type, beneficiary, target audience, health topic or technology addressed and lead organization for the development. The criteria used are described under each heading below.

Document Type

In order to be able to place guidance in the appropriate context (including when assessing quality) a distinction was made between the different categories of guidance. Each guidance was categorized as: Technical Normative or Operational or Legal/Human Rights/Ethics or Descriptive. Only one category is assigned to any one guidance.

Distinctions were as follows:

Technical **Normative Guidance** - defined as guidance that provides detailed information on what to do regarding health care interventions. It contains standards of care and gives directions on what interventions to promote, to execute, how to treat and what treatment to give. It also includes derivative products like for example a checklist for newborn care.

Operational Guidance -defined as guidance that describes in detail how to implement interventions recommended in the technical normative guidance. It includes operational manuals, tool kits, handbooks, etc. The primary audience is often the program implementer, program managers, coordinator, etc.

Descriptive Guidance - defined as guidance that provides general information but does not include in detail what to do. This includes Fact Sheets, Framework, Statement Summary, or policy documents.

Legal/Human rights/Ethics Guidance -defined as normative guidance based on principles or conventions. The guidance may not be based on hard science or evidence but on the right thing to do.

Two additional variables were assessed to determine if guidance was meant for war or conflict situations. These included:

Guidance is specifically meant for war or conflict situations if war, conflict, or a related term is mentioned in the title or introduction or described as objectives or purpose of guidance.

Guidance is applicable to war or conflict situations, if war, conflict, or a related term is mentioned in the title or introduction or described as objectives or purpose of guidance or if the document contains a section that has war, conflict, or a related term in its heading or if the reviewer otherwise considers the guidance applicable to war or conflict situations.

Guidance was also assessed if it referred to access to resources, mobility and security and disruption to health service delivery.

Guidance referred to reduced access to resources (material, personnel etc), if reduction in resources including finance, materials, personnel, capacity is explicitly referred to in the guidance.

Guidance referred to reduced mobility and security (patients or health personnel), if reduction in human (patient or staff) mobility due to safety reasons is explicitly referred to in the guidance.

Guidance referred to other disruptions in health service delivery, if disruption in the health system delivery is explicitly referred to in the guidance

<u>Beneficiary Group</u>

For each document it was noted whether substantive recommendations were offered for a specific beneficiary group. This was based on the judgement of the reviewers. Where there was disagreement this was solved in dialogue between the reviewers. The following categories were used, and are not mutually exclusive:

- Women (including maternal health and reproductive health as appropriate)
- Newborn
- Child
- Adolescent

Target Audience

For each document it was noted whether a target audience was identified explicitly. For those documents where it was explicitly mentioned, the following categories were considered, and not mutually exclusive:

- First level health worker
- Hospital Professional
- Program Manager
- Individual
- Family

This was based exclusively on self-reporting.

Health topic or technology

Reviewers noted whether a document offered substantive recommendations on any of the following categories. This is based on the judgement of the reviewers. Where there was disagreement, this was solved in dialogue between the reviewers. Key words used for categorization were noted in case further review was required.

- Sexual Health
- Pregnancy and perinatal care
- Immunization

- Infection and Communicable Disease
- Non-Communicable Disease
- Mental Health (Including early childhood development)
- Injury/trauma
- Violence
- Nutrition
- Other

Lead Organization

Reviewers identified lead organizations for the development of the guidance as indicated on the first page or acknowledgement section of the document, not simply contributing towards. If developed in a network or partnership, then the network is noted as lead, as well as the individual organizations at the highest level/leading the exercise, as noted in acknowledgements section. The Presence of logo is indication, but if in conflict with acknowledgement, the acknowledgement is followed. Guidance may be developed by only one Organization or by multiple organizations.
