# Supplementary material for: The availability of global guidance for the promotion of women’s, newborns’, children’s and adolescents’ health and nutrition in conflicts
Source: BMJ Glob Health. 2020 Nov 22;5(Suppl 1):e002060. doi: 10.1136/bmjgh-2019-002060 (PMC7684670; doi:10.1136/bmjgh-2019-002060)
Supplement: Supplementary data [file bmjgh-2019-002060supp003.pdf]

Supplemental Table 3: Key Informant Interviews.

Category	Planned Sample (Organizations)	Actual Interviews (Organizations (interviewed individuals))	Subjects
UN	WHO, UNICEF, UNFPA, UNHCR, OCHA	WHO (3), UNICEF (2), UNFPA (1), UNHCR (1), OCHA (4)	Guideline development, dissemination, emergency preparedness and response, MNCAH and nutrition
Network	IAWG<br>ENN	IWAG (1), ENN (2)	Guideline development, dissemination, SRH, nutrition
(I)NGO/Red Cross Movement	IRC, ICRC, MSF, Save the Children, World Vision	IRC (1), ICRC (1), MSF (2), Save the Children (1), World Vision (2)	Guideline development, dissemination, emergency preparedness and response, MNCAH and nutrition
Donor	ECHO	ECHO(1)	Coordination, finance, emergency preparedness and response

UN- United Nations, NGO- Non Government Organization, WHO-World Health Organization, UNICEF-United Nations Children's Fund, UNFPA- United Nations Fund for Population Activity, UNHCR- United Nations High Commissioner for Refugees, OCHA- Office for the Coordination of Humanitarian Affairs, IAWG- Interagency Working Group, ENN- Essential Nutrition Network, IRC-International Rescue Committee, ICRC-International Committee of the Red Cross, ECHO-European Civil Protection and Humanitarian Aid Operation, MSF- Medicins Sans Frontieres International
