# Supplementary material for: The availability of global guidance for the promotion of women’s, newborns’, children’s and adolescents’ health and nutrition in conflicts
Source: BMJ Glob Health. 2020 Nov 22;5(Suppl 1):e002060. doi: 10.1136/bmjgh-2019-002060 (PMC7684670; doi:10.1136/bmjgh-2019-002060)
Supplement: Supplementary data [file bmjgh-2019-002060supp004.pdf]

Supplementary table 4. Interview-guide for the in-depth interviews

**Question number**	**Question formulation**
1.	What SRMNCAH and nutrition guidance do you use or promote?
2.	Within your organization who decides on what needs to be developed and how is the decision made?
3.	What process do you follow for developing guidance and who is involved?
4.	How often do you update your guidance?
5.	How do you disseminate your guidance?
6.	How do you know if your guidance is used?
7.	From your experience what gaps exist to address nutrition guidance in humanitarian settings?
