# Supplementary material for: The availability of global guidance for the promotion of women’s, newborns’, children’s and adolescents’ health and nutrition in conflicts
Source: BMJ Glob Health. 2020 Nov 22;5(Suppl 1):e002060. doi: 10.1136/bmjgh-2019-002060 (PMC7684670; doi:10.1136/bmjgh-2019-002060)
Supplement: Supplementary data [file bmjgh-2019-002060supp005.pdf]

Supplementary table 5 - List of organizations and networks, the websites of which were searched for guidance documents (with hyperlinks to the actual websites)

Name of organization (with hyperlinks to their websites)
Action Contre la Faim
American Refugee Committee
AVSI Foundation
CARE International
Center for Reproductive Rights
Centre for Disease Control and Prevention - United States of America
Centre for Research on the Epidemiology of Disasters
Columbia University
Concern Worldwide
CORE Group
Department for International Development - Government of United Kingdom
Department of State/Bureau of Population, Refugees, and Migration – United States
Emergency and Relief Agency - Arab Medical Union
Emory University
ENN
European Commission Humanitarian Aid Office
Food and Agriculture Organization of the United Nations
Global Alliance for Improved Nutrition: Home
Global Fund to Fight HIV/AIDS, Tuberculosis and Malaria
GOAL
Helen Keller International
HelpAge International
Hope worldwide
iMMAP
Institute for Global Health - UCL
InterAction
International Centre for Migration and Health

Name of organization (with hyperlinks to their websites)
International Committee of the Red Cross
International Consortium for Emergency Contraception, hosted by Management
International Council of Nurses
International Council of Voluntary Agencies
International Federation of Red Cross and Red Crescent Societies
International Medical Corps
International Organization for Migration (IOM)
International Orthodox Christian Charities | Home
International Planned Parenthood Federation – The SPRINT Initiative
International Rescue Committee
Ipas
Jhpiego
John Snow, Inc.
Johns Hopkins University Center for Refugee & Disaster Response
Malaysian Medical Relief Society
Malteser International
Marie Stopes International
Medair
Médecins du Monde
Médecins Sans Frontières
Medical Teams International
Nutrition International: Home
Office of Foreign Disaster Assistance – United States of America
Plan International
Population Action International
Premiere Urgence Internationale
Public Health Agency of Canada
Public Health England
RedR UK
Relief International

**Name of organization (with hyperlinks to their websites)**
Research for Health in Humanitarian Crises - Elrha
Samaritan's Purse — International Relief
Save the Children (UK)
Save the Children (USA)
Sphere Project
Terre des homes
The Harvard Humanitarian Initiative
United Nations Children's Fund (UNICEF)
United Nations High Commissioner for Refugees (UNHCR)
United Nations Population Fund (UNFPA)
Université catholique de Louvain
UNSCN
Valid International
Women’s Refugee Commission
World Association for Disaster and Emergency Medicine
World Food Program (WFP)
World Health Organization (WHO)
World Vision
