# Supplementary material for: The availability of global guidance for the promotion of women’s, newborns’, children’s and adolescents’ health and nutrition in conflicts
Source: BMJ Glob Health. 2020 Nov 22;5(Suppl 1):e002060. doi: 10.1136/bmjgh-2019-002060 (PMC7684670; doi:10.1136/bmjgh-2019-002060)
Supplement: Supplementary data [file bmjgh-2019-002060supp006.pdf]

Supplementary table 6. List of documents included in the analysis

Name of document
Communicable diseases and severe food shortage
Do's and don'ts in community-based psychosocial programming in regard to sexual violence in conflict-affected settings
Guidelines for Integrating Gender-based Violence Interventions in Humanitarian Action: Reducing risk, promoting resilience and aiding recovery
Guidance Brief HIV Interventions for Young People in Humanitarian Emergencies
Integrating sexual and reproductive health into health emergency and disaster risk management
Inter-agency field manual on reproductive health in humanitarian settings *
Inter-Agency reproductive health kits for crisis situations 5th edition *
Malaria control in humanitarian emergencies - An inter-agency field handbook. Second edition
Mental health and psychosocial support for conflict-related sexual violence: principles and interventions
mhGAP Humanitarian Intervention Guide (mhGAP-HIG): Clinical Management of Mental, Neurological and Substance Use Conditions in Humanitarian Emergencies
Guidance on infant feeding and HIV in the context of refugees and displaced populations, June 2009 Version 1.1
Interim Operational Considerations for the feeding support of infants and young children under 2 years of age in Refugee and nigrant transit settings in Europe
Minimum Standards for Prevention and Response to Gender-Based Violence in Emergencies
Nutrition matters: Guidance for nutrition programming
Newborn Health in Humanitarian Settings Field Guide
Policy Statement on HIV Testing and Counselling for Refugees and other persons of concern to UNHCR
Early Childhood Development in Emergencies Integrated Programme Guide *
The Sphere Handbook *
Operational guidelines on improving newborn health in refugee operations
Vaccination in humanitarian emergencies implementation guide
Infant and Young Child Feeding in Refugee Situations: A Multi-Sectoral Framework for

Action *
Guideline for Reviewing Maternal Deaths
Violence against Women and Girls in Humanitarian Emergencies - CHASE Briefing Paper
Handbook for Coordinating Gender-based violence interventions in humanitarian settings *
Assessment of HIV in Internally Displaced Situations
IASC Guidelines for Addressing HIV in Humanitarian Settings
WASH'Nutrition
Adolescent sexual and reproductive health toolkit for humanitarian settings a companion to reproductive health in humanitarian settings
Policy Brief Shadows to Spotlight Making Adolescents Visible in Already Collected Data *
Improving Adolescent Sexual and Reproductive Health Programs in Humanitarian Settings Summary *
Disabilities among refugees and conflict-affected populations Resource kit for field workers *
Saving women's lives in refugee and other crisis situations Manual vacuum aspiration *
Anesthesia Handbook - ICRC
HIV/AIDS Field Guide: a Planning and Practice Guide to Integrating HIV/AIDS into ICRC's Health Work
Public Health Engineering in Precarious Situations
Menstrual Hygiene Management in Emergencies Toolkit
Programme Guide Early Childhood Care and Development in Emergencies
Technical Note Early Childhood Development, Nutrition and Health in Emergencies
Technical Note Early Childhood Development and Child Protection in Emergencies
Psychological first aid guide for field workers
Evaluation of CFS - Tools and guidance for monitoring and evaluating CFS
Guidelines for the selection and use of new ready-to-use-therapeutic food (RUTF) products in world vision programmes
CMAM Toolkit - Rapid start-up resources for emergency nutrition personnel
Infant and Young Child Feeding Practices Standard Operating Procedures for the Handling of Breastmilk Substitutes (BMS) in Refugee Situations for Children 0-23 Months
Women Adolescent and Young Child Spaces - Responding to women and children's needs in emergencies (Global HEA, Health, Nutrition, WASH)

WV Policy governing procurement and use of milk products in field programmes
Infant and Young Child Feeding in Emergencies - Operational Guidance for Emergency Relief Staff and Programme Managers
Guide to Maternal Newborn and Child Health and Nutrition in Emergencies
Manual Baby Friendly Spaces Holistic Approach for Pregnant, Lactating Womenand their very young children in Emergency
Manual for the health care of children in humanitarian emergencies
UNHCR Operational Guidance on the Use of Special Nutritional Products to Reduce Micronutrient Deficiencies and Malnutrition in Refugee Populations
UNICEF Programming Guide Infant and Young Child Feeding
Preventing Moderate Acute Malnutrition (MAM) through nutrition specific interventions
Guidelines for Selective Feeding - Management of Malnutrition in Emergencies
Infant and Young Child Feeding in Emergencies - Guide for Programming
Newborn Health in Emergencies Summary Sheet
Emergency contraceptive pills: fast facts for decision makers and program managers in crisis-affected settings *
Working with refugees engaged in sex work: a guidance note for humanitarians *
PMTCT in humanitarian settings - Part I Lessons learned and recommendations *
Guidelines for child friendly spaces in emergencies *
Moderate Acute Malnutrition: A Decision Tool for Emergencies *
HIV and infant feeding in emergencies: operational guidance *
Integrating Early Childhood Development (ECD) activities into Nutrition Programmes in Emergencies. Why, What and How *
A Guide to Sexual and Gender-Based Violence Legal Protection in Acute Emergencies *
Adolescent Girls: Mitigating Risk, Maximizing Potential: A Framework for Humanitarian Response *
Caring for Child Survivors of Sexual Abuse: Guidelines for health and psychosocial service providers in humanitarian settings *
Improving family planning service delivery in humanitarian crises
Including Adolescent Girls with Disabilities in Humanitarian Programs: Principles and Guidelines
Interagency Gender-Based Violence Case Management Guidelines: Providing Care and case

management Services to Gender-Based Violence Survivors in Humanitarian Settings
Minimum Standards for Child Protection in Humanitarian Action *
MISP Process Evaluation Tools (2017) *
Reporting and Interpreting Data on Sexual Violence from Conflict-Affected Countries: “Do’s and Don’ts”
Service Provision Mapping Tool: Urban Refugee Response Mapping humanitarian and host community organizations relevant to GBV prevention and GBV risk mitigation
Toolkit for Monitoring and Evaluating Gender-Based Violence Interventions along the Relief to Development Continuum
Urban Gender-Based Violence Risk Assessment Guidance: Identifying Risk Factors for Urban Refugees *

Where possible, the document titles provide a hyperlink to the actual document. Documents, that are marked with an asterisk (*) are no longer available, or have been updated or moved. Several of these documents will be available the Knowledge Bank of the Global Health Cluster (https://www.who.int/health-cluster/resources/publications/en/).
