# Supplementary material for: The availability of global guidance for the promotion of women’s, newborns’, children’s and adolescents’ health and nutrition in conflicts
Source: BMJ Glob Health. 2020 Nov 22;5(Suppl 1):e002060. doi: 10.1136/bmjgh-2019-002060 (PMC7684670; doi:10.1136/bmjgh-2019-002060)
Supplement: Supplementary data [file bmjgh-2019-002060supp007.pdf]

BMJ Publishing Group Limited (BMJ) disclaims all liability and responsibility arising from any reliance placed on this supplemental material which has been supplied by the author(s)

Supplementary table 7: Number of identified guidance documents, by the addressed health topic or technology and intended target audience

		Target Audience					
Addressed health topic/technology	Number of documents	Individual	Family	Field Level Health Worker	Hospital Professional	Programme Managers	Not mentioned
Sexual and reproductive health	18	2	0	2	1	8	8
Pregnancy and perinatal care	10	1	0	3	3	2	5
Immunizations	7	1	0	5	4	3	0
Communicable diseases and infections	36	2	1	14	11	15	9
Non-communicable diseases	3	0	0	2	1	2	0
Mental health	18	3	1	5	3	9	2
Injuries and trauma	9	0	0	6	6	1	1
Violence including sexual violence	25	8	0	6	6	12	2
Nutrition	38	3	0	9	5	26	6
Other	10	0	0	5	3	3	3
Documents addressing this target audience		12	2	31	23	48	20
