# Supplementary material for: The availability of global guidance for the promotion of women’s, newborns’, children’s and adolescents’ health and nutrition in conflicts
Source: BMJ Glob Health. 2020 Nov 22;5(Suppl 1):e002060. doi: 10.1136/bmjgh-2019-002060 (PMC7684670; doi:10.1136/bmjgh-2019-002060)
Supplement: Supplementary data [file bmjgh-2019-002060supp008.pdf]

Supplementary table 8. Mean AGREE II scores for individual questions making up the six domains of document quality

**Domain / individual question**	**Mean AGREE II score**
Scope and purpose	
1. The overall objective(s) of the guideline is (are) specific described.	6.2
2. The health question(s) covered by the guideline is (are) specifically described.	6.1
3. The population (patient, public, etc.) to whom the guideline is meant to apply is specifically described.	5.7
Stakeholder involvement	
4. The guideline development group includes individuals from all relevant professional groups.	3.8
5. The views and preferences of the target population (patients, public, etc.) have been sought.	2.9
6. The target users of the guidelines are clearly defined.	5.6
Rigor of development	
7. Systematic methods were used to search for evidence.	1.3
8. The criteria for selecting the evidence are clearly described.	1.2
9. The strengths and limitations of the body of evidence are clearly described.	1.4
10. The methods for formulating the recommendations are clearly described.	2.5
11. The health benefits, side-effects, and risks have been considered in formulating the recommendations.	4.9
12. There is an explicit link between the recommendations and the supporting evidence.	3.9
13. The guideline have been externally reviewed by experts prior to its publications.	1.9
14. A procedure for updating the guideline is provided.	2.1
Clarity of presentation	
15. The recommendations are specific and unambiguous.	5.5

Domain / individual question	Mean AGREE II score
16. The different options for management of the condition or health issue are clearly described.	4.5
17. Key recommendations are easily identifiable.	5.8
Applicability	
18. The guideline describes facilitators and barriers to its application.	3.9
19. The guideline provides advice and/or tools on how the recommendations should be put into practice.	4.5
20. The potential resource implications of applying the recommendations have been considered.	2.9
21. The guideline presents monitoring and/or auditing criteria.	3.3
Editorial independence	
22. The views of the funding body have not influenced the content of the guideline.	2.2
23. Competing interests of guideline development group members have been recorded and addressed	1.2
