# Supplementary material for: The availability of global guidance for the promotion of women’s, newborns’, children’s and adolescents’ health and nutrition in conflicts
Source: BMJ Glob Health. 2020 Nov 22;5(Suppl 1):e002060. doi: 10.1136/bmjgh-2019-002060 (PMC7684670; doi:10.1136/bmjgh-2019-002060)
Supplement: Supplementary data [file bmjgh-2019-002060supp009.pdf]

Supplementary table 9 -. Mean AGREE II scores for the six domains of document quality, by the intended beneficiary group

		**Mean AGREE II scores for the six domains of document quality**					
**Intended beneficiary group[1]**	**Number of documents**	**Scope and purpose**	**Stakeholder involvement**	**Rigor of development**	**Clarity of presentation**	**Applicability**	**Editorial independence**
Women	46	82%	49%	22%	69%	42%	9%
Newborns	22	88%	54%	25%	76%	48%	9%
Children	69	86%	56%	26%	73%	48%	13%
Adolescents	32	81%	48%	19%	61%	41%	11%
All documents together	105	84%	52%	23%	71%	45%	12%

[1]Numbers are not mutually exclusive, i.e. the same document may be considered in different rows, if it addresses several beneficiary groups
