# Supplementary material for: The availability of global guidance for the promotion of women’s, newborns’, children’s and adolescents’ health and nutrition in conflicts
Source: BMJ Glob Health. 2020 Nov 22;5(Suppl 1):e002060. doi: 10.1136/bmjgh-2019-002060 (PMC7684670; doi:10.1136/bmjgh-2019-002060)
Supplement: Supplementary data [file bmjgh-2019-002060supp010.pdf]

Supplementary table 10 - Mean AGREE II scores for the six domains of document quality, by the main health topic or technology addressed in the document

		Mean AGREE II scores for the six domains of document quality					
**Addressed health topic or technology[1]**	**Nr of documents**	**Scope and purpose**	**Stakeholder involvement**	**Rigor of development**	**Clarity of presentation**	**Applicability**	**Editorial independence**
Sexual and reproductive health	18	80%	44%	20%	67%	41%	11%
Pregnancy and perinatal care	10	81%	45%	18%	67%	31%	12%
Immunizations	7	94%	59%	18%	81%	40%	7%
Communicable diseases and infections	36	89%	51%	28%	77%	43%	11%
Non-communicable diseases	3	100%	59%	20%	94%	46%	15%
Mental health	18	80%	55%	16%	66%	40%	9%
Injuries and trauma	9	90%	61%	24%	77%	42%	19%
Violence and injuries	25	84%	58%	22%	65%	47%	14%
Nutrition	38	89%	54%	29%	76%	50%	15%
Other	10	79%	42%	18%	67%	39%	4%
All documents together	105	84%	52%	23%	71%	45%	12%

[1]Numbers are not mutually exclusive, i.e. the same document may be considered in different rows, if it addresses several health topics or technologies
